# Adherence to event‐driven HIV PrEP among men who have sex with men in Amsterdam, the Netherlands: analysis based on online diary data, 3‐monthly questionnaires and intracellular TFV‐DP

**DOI:** 10.1002/jia2.25708

**Published:** 2021-05-10

**Authors:** Vita W Jongen, Elske Hoornenborg, Mark AM van den Elshout, Anders Boyd, Hanne ML Zimmermann, Liza Coyer, Udi Davidovich, Peter L Anderson, Henry JC de Vries, M Prins, Maarten F Schim van der Loeff, the Amsterdam PrEP Project team in the HIV Transmission Elimination Amsterdam (H‐TEAM) Initiative

**Affiliations:** ^1^ Department of Infectious Diseases Public Health Service Amsterdam Amsterdam The Netherlands; ^2^ Stichting HIV Monitoring Amsterdam the Netherlands; ^3^ Department of Social Psychology University of Amsterdam Amsterdam the Netherlands; ^4^ Department of Pharmaceutical Sciences University of Colorado Anschutz Medical Campus Aurora CO USA; ^5^ Amsterdam UMC Department of Dermatology Amsterdam institute for Infection and Immunity (AII) location Academic Medical Center University of Amsterdam Amsterdam The Netherlands; ^6^ Amsterdam UMC Internal Medicine Amsterdam institute for Infection and Immunity (AII) University of Amsterdam Amsterdam The Netherlands

**Keywords:** HIV, Pre‐exposure prophylaxis, On‐demand PrEP, medication adherence, HIV prevention and control, men who have sex with men

## Abstract

**Introduction:**

Event‐driven pre‐exposure prophylaxis (edPrEP) with oral tenofovir disoproxil fumarate (TDF)/emtricitabine (FTC) is highly effective for preventing HIV acquisition in men who have sex with men (MSM) and is preferred over daily PrEP by some MSM. However, it is largely unknown how well MSM adhere to edPrEP. We then aimed to assess PrEP protection during CAS among MSM using edPrEP and participating in the Amsterdam PrEP demonstration project (AMPrEP).

**Methods:**

We analysed data from participants enrolled in AMPrEP who were taking edPrEP. We measured adherence through (1) a mobile application in which sexual behaviour and PrEP‐use were recorded daily, (2) three‐monthly self‐completed questionnaires and (3) dried blood spot (DBS) samples collected around six, twelve and twenty‐four months after PrEP initiation. We assessed the proportion of days with condomless anal sex (CAS) acts that were protected by PrEP, per partner type (i.e. steady partners, known casual partners, unknown casual partners), and the proportion of three‐month periods during which PrEP was correctly used. Intracellular TFV‐diphosphate (TFV‐DP) concentrations were determined from DBS. Good adherence was defined as at least one tablet before and one tablet within 48 hours after a CAS act.

**Results:**

Between 11 September 2015 and 6 October 2019, 182 of 376 MSM (48.4%) used edPrEP for at least one three‐month period. Of the 8224 CAS days that were reported in the app during edPrEP‐use, we observed good protection for most CAS days involving steady partners (n = 1625/2455, 66.9%), known casual partners (n = 3216/3472, 92.6%) and unknown casual partners (n = 2074/2297, 90.3%). Men reported consistently correct PrEP‐use in 851 (81.4%) of the 1046 three‐month periods of edPrEP‐use. The median TFV‐DP concentration was 591 fmol/sample (interquartile range = 270 to 896).

**Conclusions:**

Adherence to edPrEP was high as determined from the online app and questionnaire. DBS measurements were consistent with two to three tablets per week on average.

## 1 INTRODUCTION

Pre‐exposure prophylaxis (PrEP) has been proven to be highly effective against the acquisition of human immunodeficiency virus (HIV) [1, 2]. The World Health Organization has therefore recommended that PrEP be offered to people at increased risk for HIV (i.e. populations with an incidence of >3 per 100 person‐years) [3]. PrEP can be taken daily or as an event‐driven regimen, whereby two tablets are taken twenty‐four to two hours before a sex act, followed by one tablet every 24 hours until 48 hours after the last sex act [2, 4]. Since fewer tablets are required [5, 6], event‐driven PrEP (edPrEP) could reduce individual and population level costs and improve cost‐effectiveness [7, 8]. Additionally, individuals who prefer not to use PrEP daily or cannot use daily PrEP due to co‐morbidities, but who still engage in condomless anal sex (CAS), may view edPrEP as a more apposite alternative [9, 10].

Adherence is pivotal to the effectiveness of PrEP [1]. However, few studies have been conducted on adherence to edPrEP among men who have sex with men (MSM) or have compared the relationship between different adherence assessment tools [5, 11, 12, 13, 14]. Assessing adherence with questionnaires can be prone to recall bias, which might be exacerbated during variable PrEP‐use (e.g. dependent on having CAS). Adherence has also been assessed by measuring intracellular concentrations of tenofovir diphoshate (TFV‐DP) and emtricitabine triphosphate (FTC‐TP) in dried blood spots (DBS) [15, 16]. Since current adherence interpretations for TFV‐DP and FTC‐TP have been based on daily dosing, their interpretation in the setting of event‐driven use is unclear.

To gain insight into edPrEP adherence, we aimed to assess PrEP use during CAS among MSM using edPrEP and participating in the Amsterdam PrEP observational cohort study (AMPrEP). Specifically, we aimed to quantify the number of CAS acts which were poorly or not protected by PrEP, stratified by partner type, to obtain an indication of risk for HIV acquisition during CAS. We also assessed determinants of poor or no PrEP protection and variation in protection over time. Lastly, we aimed to assess whether intracellular TFV‐DP from DBS could be used to measure adherence for edPrEP users. We used data from (1) a mobile diary‐based application (app), (2) three‐monthly questionnaires and (3) DBS to study these aims.

## 2 METHODS

### 2.1 Study design and participants

The AMPrEP project was a demonstration project that aimed to assess the uptake and feasibility of daily and edPrEP among MSM and transgender persons in Amsterdam, the Netherlands. Full study procedures have been published [17]. In brief, enrolment took place between 3 August 2015 and 31 May 2016. HIV‐negative MSM and transgender persons were eligible for inclusion if they were ≥18 years old and reported any of the following in the previous six months: CAS with casual partners, at least one diagnosed bacterial sexually transmitted infection (STI), use of post‐exposure prophylaxis, or sex with a partner living with HIV with a detectable or unknown viral load. At the baseline visit, participants could choose between daily and edPrEP regimens. Participants were monitored every three months and were allowed to switch between regimens at these visits. PrEP was provided free‐of‐charge by physicians and nurses at the STI clinic of the Public Health Service of Amsterdam for all study visits. Two participants using daily PrEP became HIV‐positive during follow‐up [18]. The AMPrEP project ended December 2020; for this analysis, we used data from the first 42 months after PrEP initiation of each participant.

Written informed consent was obtained from all participants. The AMPrEP study was approved by the ethics board of the Amsterdam University Medical Centers, location Academic Medical Center, the Netherlands (NL49504.018.14) and is registered with the Netherlands trial registration (NTR5411).

### 2.2 Procedures

We developed an app for Android and iOS to collect daily information on PrEP‐use and sexual behaviour. The design and use of the app have been described elsewhere [6]. We restricted access to the app to AMPrEP participants through a personal registration code. We saved app data on a protected server at the Public Health Service of Amsterdam using unique study identifiers. Using the app was not mandatory and participants did not receive any incentive for app use. During the follow‐up period, we asked participants to answer two questions in the app on a daily basis: (1) “Did you take a pill today?” and (2) “Did you have anal sex today?”, both to be answered as “yes” or “no”. If anal sex occurred during the day, the app prompted six additional questions regarding partner type (steady partner, known casual partner and/or unknown casual partner) and whether a condom was used during the sex act(s) with these partner types. We defined a steady partner as a sex partner with whom the participant reported being in a serious relationship (independent of the length of time). We defined a known casual partner as a sex partner who was known to the participant, but with whom he was not in a relationship, and an unknown casual partner as a sex partner who was unknown to the participant. Participants could indicate multiple sex acts and partner types per day.

At baseline and three‐monthly study visits, we tested participants for HIV and STI and asked participants to complete a self‐administered computer‐assisted questionnaire on sexual behaviour and PrEP‐use, as described previously [17]. We asked participants to complete the Alcohol Use Disorder Identification test (AUDIT) [19] and Drug Use Disorder Identification Test (DUDIT) [20] every year.

For DBS, we used whole blood collected through phlebotomy conducted at three time points after PrEP initiation: three, six or nine; twelve; and twenty‐four months. Blood was spotted onto Whatman 903^TM^ Protein Saver Cards (GE Healthcare Bio‐Sciences Corp., Piscataway, NJ, USA). Blood was allowed to dry for ≥2 hours at room temperature, after which the cards were sealed in plastic bags containing a desiccant with a humidity indicator (MiniPax®, Multisorp Technologies, Buffalo, NY, USA) and stored at −20°C. DBS samples were shipped in batches for analysis at Skaggs School of Pharmacy and Pharmaceutical Sciences (University of Colorado Anschutz Medical Campus, Aurora, CO, USA). We sent 12‐ and 24‐month samples and if the former sample was unavailable, we sent the 3‐, 6‐ or 9‐month sample instead. The procedure to measure TFV‐DP and FTC‐TP in DBS has been described previously [21]. Lower limit of quantification was 25 and 100 fmol/sample for TFV‐DP and FTC‐TP, respectively. We did not communicate intracellular TFV‐DP and FTC‐TP results to participants.

### 2.3 Outcomes

Based on data recorded in the app, we evaluated PrEP protection per day on which CAS was reported (henceforth “CAS days”). We categorized edPrEP protection as excellent, good, poor or none (Table 1). If no data were recorded on one or more CAS days, we assumed no PrEP was used on those days.

**Table 1:** Definitions of PrEP protection^a^

Day of CAS or day before CAS	Pills taken on		
	First day after CAS	Second day after CAS	Conclusion regarding PrEP protection
+	+	+	Excellent
+	+	−	Good
+	−	+	Good
+	−	−	Poor
−	+	−	Poor
−	−	+	Poor
−	+	+	Poor
−	−	−	No

CAS, condomless anal sex; PrEP, pre‐exposure prophylaxis; +, pill taken at given moment; −, pill not taken at given moment.

^a^ Definitions based on Molina *et al*. [2]

Based on data from questionnaires, we evaluated PrEP protection as consistently correct edPrEP‐use (defined as two pills twenty‐four to two hours before a sex act, followed by one tablet every 24 hours until 48 hours after the last sex act [22]; henceforth “correct PrEP‐use”) during three‐month follow‐up periods.

Based on DBS samples from participants using edPrEP in the past three months, we estimated TFV‐DP concentrations in those who reported CAS days in the app during the six weeks before DBS collection. We assessed correlations between TFV‐DP and the number CAS days and pills taken from the app and questionnaire data. We assessed the proportion of men with detectable FTC‐DP concentrations, which indicates PrEP use in the preceding 48 hours.

### 2.4 Statistical analysis

We included all participants who reported being on edPrEP for at least one three‐month period and recorded data at least once in the app or completed a questionnaire. Baseline was defined as the date of PrEP initiation. Follow‐up started at baseline and continued until the end of the last period of edPrEP‐use or 42 months after PrEP initiation, whichever occurred first. Of participants who switched between regimens, only periods of edPrEP‐use were included in the analysis.

We compared baseline demographics of edPrEP users who were included versus excluded from analysis using rank‐sum tests for continuous variables and Pearson’s χ^2^ or Fisher’s exact tests for categorical variables. App use by study month since PrEP initiation was plotted, where we defined a study month as a period of 30 consecutive days (Analysis S1 and Table S1 for an analysis on determinants of app use).

Using app data, we assessed PrEP protection per CAS day according to partner type (steady partner, known casual partner, unknown casual partner), which was dichotomized into no/poor and good/excellent coverage. We estimated odds ratios (ORs) comparing odds of no/poor PrEP protection across levels of determinants and their 95% confidence intervals (CIs) using logistic regression with a random‐intercept to account for between‐participant variability at baseline. We forced follow‐up time and time‐updated age, and included all determinants with a *p* < 0.2 from a Wald χ^2^ test in univariable analysis and with stable estimates (i.e. standard error being smaller than the regression coefficient) in an initial multivariable model. We sequentially removed all determinants with a *p* < 0.05 to arrive at a final multivariable model. We assessed model fit using a Pearson’s χ^2^ goodness‐of‐fit test. In a sensitivity analysis, we assessed PrEP protection among participants who reported data in the app ≥90% of days per month (henceforth “high app usage”). We also assessed comparability between app and questionnaire data (Analysis S2 and Table S2).

Undetectable TFV‐DP were assigned half the value of the lower limit of quantification. We plotted log‐transformed TFV‐DP against the log‐transformed number of PrEP pills and log‐transformed number of CAS acts in the preceding six weeks, as reported in the app. In addition, we plotted log‐transformed TFV‐DP concentrations against the number of pills taken, as reported in the questionnaire, in the preceding 30 days. We regressed log‐transformed TFV‐DP concentrations on log‐transformed number of PrEP pills used, from the app and questionnaire, and log‐transformed number of CAS acts, from the app, using tobit models that account for left‐censored concentrations, while including a random‐intercept for participants. For FTC‐TP concentrations, we assessed the proportion of DBS samples with detectable FTC‐TP. In a sensitivity analysis, we restricted analysis to participants with high app usage before DBS‐collection.

We carried out analyses using Stata (v15.1, StataCorp, College Station, TX, USA).

### 2.5 Role of the funding source

The funders of this study had no role in the study design, data collection, data analysis, data interpretation and writing of the manuscript. All authors had full access to the data used in this study. The last author had final responsibility for the decision to submit for publication.

## 3 RESULTS

**Figure 1:**
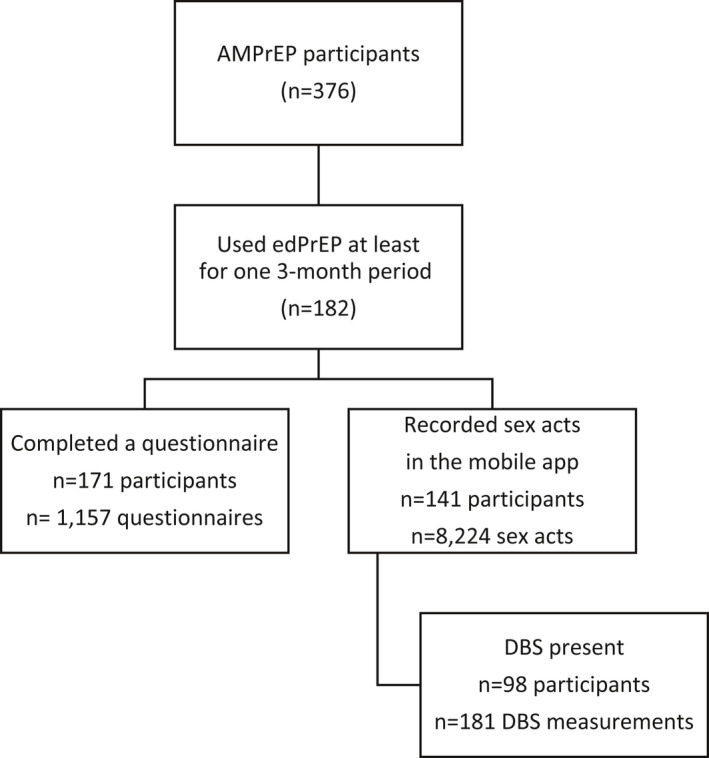
Flowchart of inclusion of participants into the analyses of questionnaire data, app data and DBS, AMPrEP observational cohort study, September 2015 to October 2019, Amsterdam, the Netherlands. app, application; DBS, dried blood spots; edPrEP, event‐driven PrEP.

Between 11 September 2015 and 6 October 2019, 182 of the 376 AMPrEP participants (48.4%) used edPrEP during at least one three‐month period (Figure 1). No HIV infections were diagnosed during edPrEP. Of the 182 edPrEP users, 41 (22.5%) did not record any data in the app (Table S3). These individuals had a lower monthly net income (*p* = 0.0114), were less often in a steady relationship (*p* = 0.0152), and more often identified as non‐exclusively homosexual (*p* = 0.0345) compared to those recording app data. Baseline sexual behaviour did not differ between app users and non‐users. The majority of edPrEP users continued recording data in the app at least once a month until 42 months after PrEP initiation (Figure S1).

**Figure 2:**
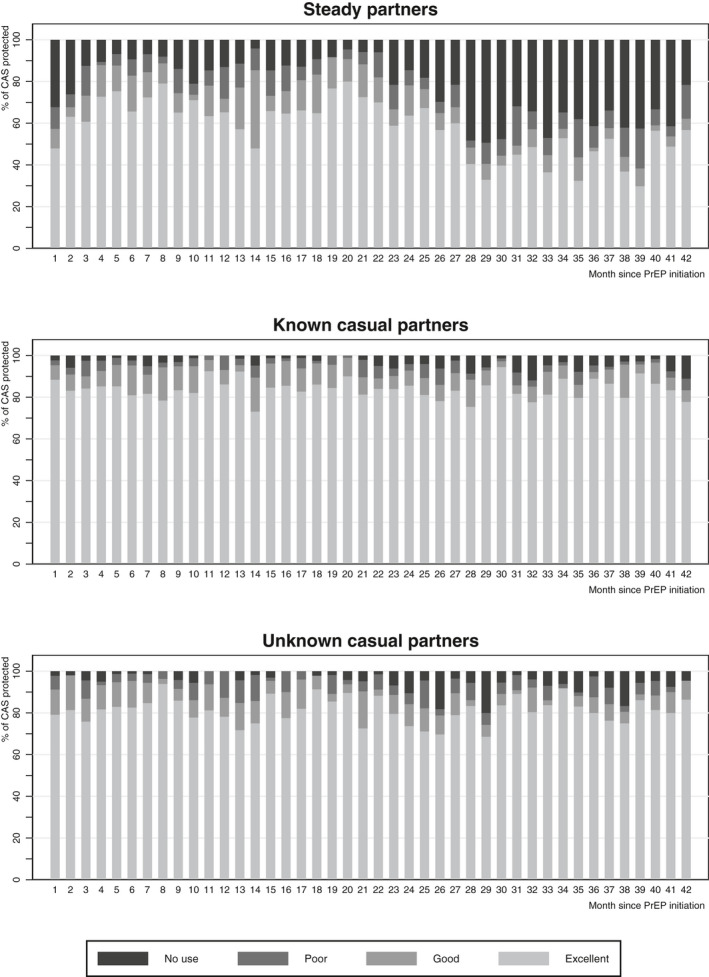
PrEP protection of condomless anal sex acts (CAS) per partner type, as reported in the AMPrEP mobile diary app, AMPrEP observational cohort study, September 2015 to October 2019, Amsterdam, the Netherlands.

In the app, 2455 (29.9%) CAS days with steady partners, 3472 (42.1%) with known casual partners, and 2297 (27.9%) with unknown casual partners were reported. Excellent PrEP protection was observed in the majority of CAS days with steady partners (56.9%), known casual partners (84.0%) and unknown casual partners (81.6%) (Table 2). We observed similar results in individuals with high app usage (Table S4). Over time, there were substantial fluctuations in edPrEP protection, notably concerning CAS days with steady partners (Figure 2).

**Table 2:** PrEP protection of condomless anal sex acts by edPrEP users per partner type as reported in the daily app, AMPrEP observational cohort study, September 2015 to October 2019, Amsterdam, the Netherlands

	Number (%) of condomless anal sex acts with					
	Steady partners (n = 2455)		Known casual partners (n = 3472)		Unknown casual partners(n = 2297)	
	n	%	n	%	n	%
PrEP protection^a^						
None	591	24.1%	126	3.6%	96	4.2%
Poor	221	9.0%	130	3.7%	127	5.5%
Good	246	10.0%	300	8.6%	200	8.7%
Excellent	1397	56.9%	2916	84.0%	1874	81.6%

PrEP, pre‐exposure prophylaxis.

^a^ Definitions of PrEP coverage in Table 1.

Univariable analysis of determinants of no/poor PrEP protection is shown in Table S5. In multivariable analysis (Table 3), there were increases over time in the odds of poor/no protection for CAS days with steady partners (aOR = 1.02 per month, 95% CI = 1.00 to 1.03) and known casual partners (aOR = 1.03 per month, 95% CI = 1.01 to 1.04). Poor/no protection was associated with the absence of drug‐use disorder (for CAS days with steady partners: aOR = 0.46, 95% CI = 0.25 to 0.85), with indication of alcohol‐use disorder (for CAS days with known casual partners: aOR = 2.21, 95% CI = 1.17 to 4.16), and with age 35 to 44 (known casual partners: aOR = 0.26, 95% CI = 0.11 to 0.61, unknown casual partners: aOR = 0.39, 95% CI = 0.17 to 0.86) and age ≥45 (known casual partners: aOR = 0.29, 95% CI = 0.13 to 0.66), unknown casual partners: aOR = 0.41, 95% CI = 0.17 to 1.00) compared to <35 years old. Model fit was good for all multivariable models (steady partners: *p* = 0.98; known casual partners: *p* = 0.99; unknown casual partners: *p* = 0.97).

**Table 3:** Determinants of poor/no protection to PrEP per sex partner type among event‐driven PrEP users (n = 141); results of multivariable multi‐level logistic regression, AMPrEP observational cohort study, September 2015 to October 2019, Amsterdam, the Netherlands

	CAS days with steady partners (n = 2455)			CAS days with known casual partners (n = 3472)			CAS days with unknown casual partners (n = 2297)		
	aOR^a^	95% CI	*p*‐value	aOR^a^	95% CI	*p*‐value	aOR^a^	95% CI	*p*‐value
Months since PrEP initiation	1.02	(1.01 to 1.03)	0.0356	1.03	(1.01 to 1.04)	0.0003	1.01	(0.995 to 1.03)	0.147
Age^b^									
<35 years	REF			REF			REF		
35 to 44 years	0.45	(0.11 to 1.82)	0.263	0.26	(0.11 to 0.61)	0.0021	0.39	(0.17 to 0.86)	0.0202
≥45 years	0.31	(0.08 to 1.27)	0.103	0.29	(0.13 to 0.66)	0.0030	0.41	(0.17 to 0.999)	0.0496
Alcohol use disorder identification test (AUDIT)^b^, ^c^									
Score <8				REF					
Score ≥8				2.21	(1.17 to 4.16)	0.0144			
Drug use disorder identification test (DUDIT)^b^, ^c^									
Score <8	REF								
Score ≥8	0.46	(0.25 to 0.85)	0.0134						

AMPrEP, Amsterdam PrEP demonstration project; CAS, condomless anal sex; CI, confidence interval; OR, odds ratio; PrEP, pre‐exposure prophylaxis.

^a^ All variables with odds ratios listed in the table were included in the final model

^b^ time‐updated

^c^ a score of eight or higher indicates the possible presence of alcohol‐ or drug‐use disorder.

Of the 182 PrEP users who used edPrEP during at least one three‐month period, 171 (94.0%) completed at least one questionnaire (Figure 1). These individuals completed 1157 questionnaires [median per participant, 6, interquartile range (IQR) = 2 to 11], of which 1046 (90.4%) indicated any use of edPrEP in the three months prior (Table 4). During these periods, an edPrEP course was started a median six times (IQR = 3 to 9), of which a median two times (IQR = 1 to 3) were not followed by CAS. The median number of reported CAS acts per three months with steady partners was 0 (IQR 0 to 5), with known casual partners 2 (IQR 0 to 6), and with unknown casual partners 1 (IQR 0 to 6).

**Table 4:** Characteristics of PrEP‐use among event‐driven PrEP users. Data from 1157 quarterly questionnaires completed by 171^a^ participants. AMPrEP observational cohort study, September 2015 to October 2019, Amsterdam, the Netherlands

	3‐month periods (n = 1157)	
	n^b^	%^b^
Number of questionnaires completed, median [IQR]	6	[2 to 11]
Started a course of PrEP at least once (3 M)		
Yes	1046	90.4 %
Number of times a course of PrEP was started (3 M), median [IQR]	6	[3 to 9]
Number of times a course of PrEP was started, after which no CAS occurred (3 M), median [IQR]^c^	2	[1 to 3]
Number of pills taken (30D)^d^, median [IQR]	12	[6 to 19]
Consistent correct PrEP‐use (3 M)^d^		
No	195	18.6%
Yes	851	81.4%
PrEP dose most often not taken when CAS was reported (3 M)^g^		
Pills before sex	47	24.1%
First pill after sex	44	22.6%
Second pill after sex	61	31.3%
Other	17	8.7%
Missing	26	13.3%
Reason for not using PrEP the last time CAS was reported (3 M)^g^		
Forgot	93	47.7%
Pills not available	19	9.7%
Lost pills	2	1.0%
Did not feel like taking PrEP	5	2.6%
Participant felt opposed to taking PrEP	2	1.0%
Unplanned sex	8	4.1%
No sex occurred	12	6.2%
Participant estimated the CAS act carried no risk	15	7.7%
Other	33	16.9%
Missing	6	3.1%
Number of sex partners (3 M), median [IQR]	8	[3 to 16]
Number of steady partners with whom CAS was reported (3 M), median [IQR]	0	[0 to 5]
Number of known casual partners with whom CAS was reported (3 M), median [IQR]	2	[0 to 6]
Number of unknown casual partners with whom CAS was reported (3 M), median [IQR]	1	[0 to 6]

Data were missing for number of pills taken (n = 20), known casual partners with whom CAS was reported (n = 21), steady partners with whom CAS was reported (n = 12). AMPrEP, Amsterdam PrEP demonstration project; CAS, condomless anal sex act; IQR, interquartile range; PrEP, pre‐exposure prophylaxis.

^a^ 11 participants did not complete a questionnaire for event‐driven PrEP during follow‐up

^b^ unless stated otherwise

^c^ of times participants indicated starting PrEP at least once in the past three months (n = 914)

^d^ in the 30 days before the questionnaire

^e^ two tablets 24 to 2 hours before a sex act, followed by a tablet every 24 hours until 48 hours have expired since the last sex act

^f^ during three‐month periods where a course of PrEP was started at least once

^g^ of three‐month periods when PrEP was not always used according to the Dutch national guidelines (n = 168).

Of the 1046 three‐month periods during which edPrEP was used, consistently correct edPrEP‐use was reported in 851 (81.4%). When asked which pill(s) they forgot most often, 24.1% of men mentioned the pills before sex, 22.6% the first pill after sex and 31.3% the second pill after sex.

One hundred and eighty‐one DBS‐measurements from 98 individuals were collected after periods of edPrEP‐use (Figure 1). The median TFV‐DP concentration was 590.5 (IQR = 270.1 to 895.1) fmol/punch and 11 (6.1%) TFV‐DP measurements were below the lower limit of quantification. FTC‐TP concentrations were below the lower limit of quantification in 124 (68.5%) DBS‐measurements, representing no dose within 48 hours. In participants with high app usage before DBS‐measurements (n = 120 samples), the median TFV‐DP concentration was slightly higher (635.7 fmol/punch, IQR = 307.4 to 989.7) and FTC‐DP concentrations were below the lower limit of quantification in 79 (65.8%) DBS‐measurements. During the six weeks before the 181 DBS‐measurements a median of 10 (IQR = 5 to 17]) pills were taken, and CAS reported a median of 3 (IQR = 1 to 5) days, according to app data. According to the questionnaire, a median of 13 (IQR = 7 to 18) pills was taken in the 30 days before DBS‐measurement.

**Figure 3:**
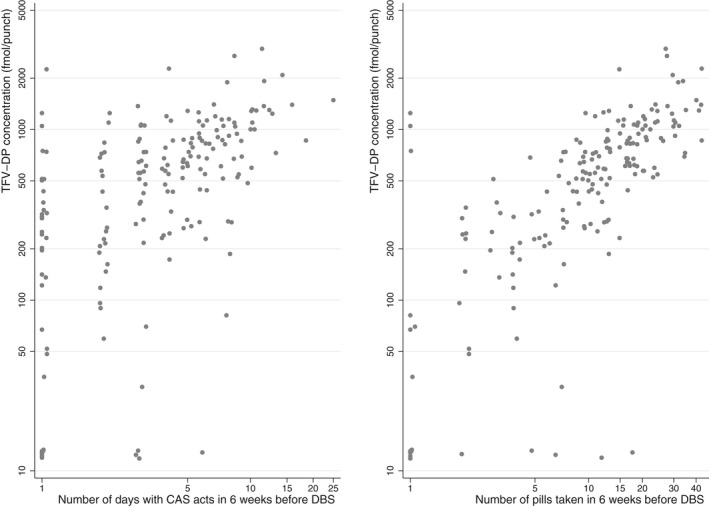
Association between log‐transformed TFV‐DP from dried blood spots (DBS) and log‐transformed condomless anal sex (CAS) days or pill use reported in the AMPrEP mobile diary app, AMPrEP observational cohort study, September 2015 to October 2019, Amsterdam, the Netherlands. Pill use and condomless anal sex days were transformed as follows: ln(number of days on which a pill was taken + 1) and ln(number of CAS days + 1).

TFV‐DP concentrations increased significantly with the number of CAS days (*p* < 0.0001) and pills used (*p* < 0.0001) reported during the six weeks before DBS‐measurements in the app (Figure 3), and with PrEP‐use reported during the 30 days before DBS‐measurements in the questionnaire (*p* < 0.0001, Figure S2). Similar results were observed in analyses limited to individuals with high app usage before DBS‐measurements (data not shown).

## 4 DISCUSSION

Using different sources of data, we provide comprehensive insight into long‐term adherence to edPrEP among MSM. Data from the daily app showed that over 90% of CAS days with known or unknown casual partners had good or excellent PrEP protection, in comparison to almost 70% of CAS days with steady partners. Data from three‐monthly questionnaires revealed that in over 80% of the questionnaire time periods, participants reported correct use of edPrEP. Furthermore, TFV‐DP concentrations obtained from DBS were positively correlated with the number of pills used and the number of CAS days in the six weeks before DBS‐measurement.

High percentages of CAS days with casual partners that were protected by PrEP have been previously observed in the Belgian Be‐PrEP‐ared study using a comparable app [11]. Levels of adherence to edPrEP have also been found to be high in the Ipergay study, but were defined differently, that is PrEP‐use for the most recent sexual act within two‐month periods [5, 14]. Conversely, lower proportions of protected sex acts with edPrEP were apparent in a study among MSM and transgender women in Thailand and the United States [12, 13]; however, these particular studies made no distinction in adherence per partner type and participants were assigned to, rather than chose, their PrEP regimen [12, 13]. Perhaps the higher levels of adherence to edPrEP in our study were because participants were free to choose their preferred regimen every three months, enabling them to comply to a PrEP regimen meeting their current needs [17].

There was a higher PrEP protection during CAS days with known and unknown casual partners compared to steady partners, as observed by others [11, 23]. Low perceived HIV risk has been identified as a major driver for no condom or PrEP‐use in a mixed‐methods study from our research group [24]. Assuming that edPrEP users perceive CAS with their steady partners as bearing lower HIV‐risk, lower edPrEP protection with these partners would be expected. This result could be concerning given that a previous modelling study suggested that a large proportion of HIV transmissions among MSM are due to CAS with steady partners [25]; however, that study was conducted in the 2000s and might not reflect the current epidemiology of HIV. When examining the determinants of PrEP protection, we found that participants <35 years old and those with an indication for alcohol‐use disorder were more likely to have no/poor PrEP protection during CAS days with known and unknown casual partners. Previous studies have reported conflicting associations between alcohol use and PrEP adherence [13, 26, 27]. Interestingly, no/poor PrEP protection during CAS days with steady partners was less common among those with an indication for drug‐use disorder. This seemingly counterintuitive finding could be explained by the planning required for drug use in sexualized settings, which likely includes PrEP‐use [28]. Moreover, the questionnaires showed that during three‐monthly periods of event‐driven PrEP use, individuals started PrEP a median two times after which no CAS occurred. This finding suggests the ability to flexibly plan PrEP use among edPrEP users. Conversely, when a PrEP dose was missed, the most common reason given was forgetting to take the pill, which may reflect that planning abilities can be compromised in the “heat of the moment” or because routine use of edPrEP has not yet been established. Over time, there were fluctuations in protection across all partner types, with the most substantial changes observed with steady partners. EdPrEP protection decreased over time during CAS days with known casual partners, although at the end of the study period over 80% CAS days with known casual partners were still protected by good/excellent PrEP‐use. Additional (e.g. online behavioural interventions [29]) and ongoing support for adherence seems warranted then for some edPrEP users.

In daily PrEP users, a TFV‐DP concentration from DBS of ≥700 fmol/punch represents an effective level in preventing most HIV infections [15, 16]. With a median TFV‐DP concentration of 590.5 fmol/punch in our study of edPrEP users, well over half of DBS measurements had an insufficient concentration based on this threshold. However, due to the sex‐act dependent nature of edPrEP, TFV‐DP concentrations measured during periods when participants have fewer sex acts are expected to be low. We showed that TFV‐DP concentrations were associated with the number of CAS acts, suggesting that individuals are using edPrEP as needed (i.e. self‐efficacy). Others have argued that lower TFV‐DP concentrations in edPrEP users might be a proxy for increased HIV susceptibility [30]. However, edPrEP users who adequately protect sex acts, but only have few acts in the preceding two months, would have good adherence despite low TFV‐DP concentrations, commensurate with the average number of doses ingested in the preceding six weeks. Therefore, the number of expected PrEP doses in the preceding six weeks must be considered when interpreting TFV‐DP concentrations in the setting of edPrEP.

Our study has some limitations. First, our data relied on participants completing the questions in the app and 22.5% of edPrEP users never filled in any of the questions. There were no differences in baseline sexual behaviour between app users and never users, albeit some characteristics that may influence sexual behaviour and PrEP use (i.e. income, relationship status at baseline and sexual preference) did differ. Second, we saw a decrease over time in the number of reported sex days in the app, which may have influenced changes in adherence over time. Third, as this study was the first PrEP demonstration study in the Netherlands, participants were likely to be more highly motivated to use PrEP; and they were mostly white, middle‐aged and highly‐educated. Thus, studies on adherence to edPrEP in other settings are needed to assess whether our results may be generalized to the broader MSM population. Fourth, statistical power may not have been sufficient to establish significance for some risk‐factors. Lastly, edPrEP usage is nuanced and quantitative data cannot fully explain why PrEP users at times use edPrEP suboptimally. Qualitative studies are needed to provide more insight into this and further explore possible areas for interventions.

## 5 CONCLUSIONS

In this PrEP demonstration project, the large majority of CAS days was protected by good or excellent adherence to edPrEP, especially CAS days with known and unknown casual partners. The difference in PrEP protection between partner types could be a reflection of PrEP‐use as it aligns with perceived HIV risk. Given that the low observed TFV‐DP concentrations are the result of fewer pills taken, adherence interpretations must be customized for edPrEP. DBS can be useful as a research tool, but its use in monitoring adherence to edPrEP in clinical practice may be limited.

## Competing interests

The Public Health Service of Amsterdam received the drugs for the Amsterdam PrEP study from Gilead Sciences based on an unconditional grant. UD received unrestricted research grants and speaker’s fees from Gilead Sciences, paid to his institute. PLA received research grants and personal fees from Gilead Sciences. HJCdV received grants from Medigene, and advisory board and speaker fees from Gilead Sciences, Medigene, Abbvie, Janssen‐Cilag and Willpharma, all paid to his institute. MP received unrestricted research grants and speaker’s fees from Gilead Sciences, Roche, Abbvie and MSD, all paid to her institute. All other authors declare no competing interest.

## Author’s contributions

EH, MP, HJVdV, UD and MFSvdL conceptualized and designed the AMPrEP demonstration project. VJ, EH, MvdE and MFSvdL contributed to the study design of this study. VJ, EH, MvdE, AB, HZ, LC, UD, PA, HJVdV, MP and MFSvdL were involved in data acquisition, data analysis or interpretation of the data. VJ drafted the manuscript. All authors read and approved the final manuscript.

## Data sharing

The AMPrEP data are owned by the Public Health Service of Amsterdam. Original data can be requested by submitting a study proposal to the steering committee of AMPrEP. The proposal format can be obtained from amprep@ggd.amsterdam.nl. Request for further information can also be submitted through the same email address. The AMPrEP steering committee verifies each proposal for compatibility with general objectives, ethical approval and informed consent forms of the AMPrEP study, and potential overlap with ongoing studies. There are no restrictions to obtaining the data and all data requests will be processed in a similar way.

## Supporting information


**Analysis S1**. Determinants of monthly app use.
**Analysis S2**. Comparability of app and questionnaire data.
**Table S1**. Determinants of monthly app use (1 to 26 days vs 0 days and use of at least 27 days vs 0 days). Results of univariable and multivariable multinomial logistic regression analysis, AMPrEP observational cohort study, September 2015 to October 2019, Amsterdam, the Netherlands
**Table S2**. Comparison of the numbers of PrEP pills taken and unknown casual partners reported in the AMPrEP mobile diary app and the 3‐monthly questionnaires, AMPrEP observational cohort study, September 2015 to October 2019, Amsterdam, the Netherlands
**Table S3**. Socio‐demographic and sexual behaviour characteristics at baseline of event‐driven PrEP users who ever used the app (n = 141) and never used the app (n = 41), AMPrEP observational cohort study, August 2015 to June 2016, Amsterdam, the Netherlands
**Table S4**. PrEP protection of condomless anal sex acts by edPrEP users per partner type as reported in the daily app by participants who reported data in the app at least 27 days within a month (n = 108), AMPrEP observational cohort study, September 2015 to October 2019, Amsterdam, the Netherlands
**Table S5**. Determinants of poor/no adherence to PrEP per sex partner type among event‐driven PrEP users (n = 141); results of univariable multi‐level logistic regression, AMPrEP observational cohort study, Septebmber 2015 to October 2019, Amsterdam, the Netherlands
**Figure S1**. App use after PrEP initiation among event‐driven PrEP users (n = 182), AMPrEP observational cohort study, September 2015 to October 2019, Amsterdam, the Netherlands.
**Figure S2**. Association between log transformed TFV‐DP concentrations from dried blood spots (DBS) and log transformed number of pills taken as reported in the 3‐monthly questionnaires, AMPrEP observational cohort study, September 2015 to October 2019, Amsterdam, the Netherlands. Number of pills was transformed as follows: ln(1+number of pills taken).
