# Supplementary material for: Adherence to event‐driven HIV PrEP among men who have sex with men in Amsterdam, the Netherlands: analysis based on online diary data, 3‐monthly questionnaires and intracellular TFV‐DP
Source: J Int AIDS Soc. 2021 May 10;24(5):e25708. doi: 10.1002/jia2.25708 (PMC8110892; doi:10.1002/jia2.25708)
Supplement: Supplementary file 1 — Analysis S1. Determinants of monthly app use. Analysis S2. Comparability of app and questionnaire data. Table S1. Determinants of monthly app use (1 to 26 days vs 0 days and use of at least 27 days vs 0 days). Results of univariable and multivariable multinomial logistic regression analysis, AMPrEP observational cohort study, September 2015 to October 2019, Amsterdam, the Netherlands Table S2. Comparison of the numbers of PrEP pills taken and unknown casual partners reported in the AMPrEP mobile diary app and the 3‐monthly questionnaires, AMPrEP observational cohort study, September 2015 to October 2019, Amsterdam, the Netherlands Table S3. Socio‐demographic and sexual behaviour characteristics at baseline of event‐driven PrEP users who ever used the app (n = 141) and never used the app (n = 41), AMPrEP observational cohort study, August 2015 to June 2016, Amsterdam, the Netherlands Table S4. PrEP protection of condomless anal sex acts by edPrEP users per partner type as reported in the daily app by participants who reported data in the app at least 27 days within a month (n = 108), AMPrEP observational cohort study, September 2015 to October 2019, Amsterdam, the Netherlands Table S5. Determinants of poor/no adherence to PrEP per sex partner type among event‐driven PrEP users (n = 141); results of univariable multi‐level logistic regression, AMPrEP observational cohort study, Septebmber 2015 to October 2019, Amsterdam, the Netherlands Figure S1. App use after PrEP initiation among event‐driven PrEP users (n = 182), AMPrEP observational cohort study, September 2015 to October 2019, Amsterdam, the Netherlands. Figure S2. Association between log transformed TFV‐DP concentrations from dried blood spots (DBS) and log transformed number of pills taken as reported in the 3‐monthly questionnaires, AMPrEP observational cohort study, September 2015 to October 2019, Amsterdam, the Netherlands. Number of pills was transformed as follows: ln(1+number of pills taken). [file JIA2-24-e25708-s001.docx]

**SupplemenT to:**

**Adherence to event-driven HIV PrEP among men who have sex with men in Amsterdam, the Netherlands: analysis based on online diary data, 3-monthly questionnaires and intracellular TFV-DP**

Vita W. Jongen, Elske Hoornenborg, Mark A.M. van den Elshout, Anders Boyd, Hanne M.L. Zimmermann, Liza Coyer, Udi Davidovich, Peter L. Anderson, Henry J.C. de Vries, M. Prins, Maarten F. Schim van der Loeff, on behalf of the Amsterdam PrEP Project team in the HIV Transmission Elimination Amsterdam (H-TEAM) Initiative.

**Supplementary Analysis 1 - Determinants of monthly app use**

*Statistical analysis*

To assess if some participants were more prone to using the app, we assessed determinants of app use (i.e. ≥27 days per month, 1-26 days per month, 0 days per month) using multinomial logistic regression.

*Results*

See Supplementary Table 1

**Supplementary Analysis 2 – Comparability of app and questionnaire data**

*Statistical analysis*

To assess comparability between the app and questionnaire data, we used a Wilcoxon signed-rank test to compare the distribution of the number of pills and unknown casual partners reported in the app and in the 3-monthly questionnaires among participants with high app usage. Because the app recorded days on which pills were taken rather than the number of pills taken, for this analysis we added one pill for every time a PrEP course was started. As the questionnaire asked about the number of pills used in the preceding 30 days and about unknown casual partners in the preceding 90 days, we assessed the number of pills recorded in the app in the same time frame.

*Results*

When comparing the questionnaire to the app data, the reported number of pills used in the preceding 30 days was higher in the questionnaire for most periods, although not significantly in all periods (Supplementary Table 2). Furthermore, the number of unknown casual partners reported in the questionnaire was significantly higher in the majority of 3-month periods.

*Interpretation*

Participants reported a higher median number of PrEP pills and unknown casual partners in the questionnaire than in the app. We previously reported that outcomes reported in app and questionnaire were highly comparable [1]; that earlier analysis only included participants who reported data in the app >90% of days every month in the first 6 months of AMPrEP. As use of PrEP taken daily is expected to be consistent, in contrast to use of edPrEP where month-to-month fluctuations occur depending on sexual behaviour, an app recording use on a daily basis may capture inconsistent PrEP-use more accurately than periodic questionnaires relying on longer recall periods. Indeed, TFV-DP concentrations were ≥900 fmol/punch in approximately 25% of participants, indicating average use of at least 4-6 tablets per week [2]. Based on the questionnaire data one would have expected a much higher proportion. Nevertheless, regular app use would be assumedly required if recall bias were to be minimized.

*References*

1. Finkenflugel RNN, Hoornenborg E, Achterbergh RCA, et al. A mobile application to collect daily data on pre-exposure prophylaxis adherence and sexual behaviour among men who have sex with men: use over time and comparability with conventional data collection. Sex Transm Dis **2019**; 46:400-6.

2. Grant RM, Anderson PL, McMahan V, et al. An observational study of preexposure prophylaxis uptake, sexual practices, and HIV incidence among men and transgender women who have sex with men. Lancet Infect Dis **2014**; 14:820-9.

**Supplementary Table 1: Determinants of monthly app use (1-26 days vs 0 days and use of at least 27 days vs 0 days). Results of univariable and multivariable multinomial logistic regression analysis, AMPrEP observational cohort study, September 2015 to October 2019, Amsterdam, the Netherlands.**

|  | **Univariable multinomial logistic regression**  **1-26 days vs 0 days** | | **Univariable multinomial logistic regression**  **≥27 days vs 0 days** | |  | **Multivariable multinomial logistic regression**  **1-26 days vs 0 days** | | | **Multivariable multinomial logistic regression**  **≥27 days vs 0 days** | | |
| --- | --- | --- | --- | --- | --- | --- | --- | --- | --- | --- | --- |
|  | *OR* | *95% CI* | *OR* | *95% CI* | *Overall p-value* | *aOR* | *95% CI* | *p-value* | *aOR* | *95% CI* | *p-value* |
| **Months since PrEP initiation** | **0.97** | **(0.95-0.98)** | **0.98** | **(0.97-0.99)** | **0.0006** | **0.97** | **(0.95-0.98)** | **0.0001** | **0.98** | **(0.97-0.99)** | **0.0070** |
| **Age, per 10 year increase^1^** | 0.89 | (0.64-1.23) | 1.24 | (0.91-1.71) | 0.083 |  |  |  |  |  |  |
| **Age^1^** |  |  |  |  |  |  |  |  |  |  |  |
| < 35 year | REF |  | REF |  | 0.151 |  |  |  |  |  |  |
| 35 - 44 year | 0.89 | (0.35-2.22) | 0.64 | (0.26-1.59) |  |  |  |  |  |  |  |
| ≥ 45 year | 0.89 | (0.36-2.20) | 1.62 | (0.70-3.75) |  |  |  |  |  |  |  |
| **Self-declared ethnicity^2^** |  |  |  |  |  |  |  |  |  |  |  |
| White | REF |  | REF |  | 0.288 |  |  |  |  |  |  |
| Non-white | 0.89 | (0.37-2.13) | 0.44 | (0.15-1.25) |  |  |  |  |  |  |  |
| **Residence^2^** |  |  |  |  |  |  |  |  |  |  |  |
| Amsterdam | REF |  | REF |  | 0.611 |  |  |  |  |  |  |
| Other | 1.47 | (0.68-3.20) | 1.27 | (0.60-2.66) |  |  |  |  |  |  |  |
| **Highest education level^2^** |  |  |  |  |  |  |  |  |  |  |  |
| No college/university | REF |  | REF |  | 0.909 |  |  |  |  |  |  |
| College/university | 1.20 | (0.43-3.36) | 1.19 | (0.49-2.88) |  |  |  |  |  |  |  |
| **Employment^2^** |  |  |  |  |  |  |  |  |  |  |  |
| Employed | REF |  | REF |  | 0.062 |  |  |  |  |  |  |
| Unemployed | 0.42 | (0.17-1.03) | 0.43 | (0.20-0.95) |  |  |  |  |  |  |  |
| **Monthly net income^2^** |  |  |  |  |  |  |  |  |  |  |  |
| Low (≤€1700) | REF |  | REF |  | 0.281 |  |  |  |  |  |  |
| Middle (€1701 to €2950) | 1.47 | (0.58-3.75) | 1.66 | (0.71-3.88) |  |  |  |  |  |  |  |
| High (>€2950) | 2.17 | (0.84-5.60) | 2.76 | (1.10-6.92) |  |  |  |  |  |  |  |
| **Steady relationship^2^** |  |  |  |  |  |  |  |  |  |  |  |
| No | REF |  | REF |  | 0.304 |  |  |  |  |  |  |
| Yes | 1.21 | (0.55-2.67) | 1.76 | (0.85-3.63) |  |  |  |  |  |  |  |
| **Living situation^2^** |  |  |  |  |  |  |  |  |  |  |  |
| Alone | REF |  | REF |  | 0.074 |  |  |  |  |  |  |
| With partner | 1.06 | (0.38-2.92) | 2.13 | (0.90-5.04) |  |  |  |  |  |  |  |
| With others | 1.48 | (0.56-3.94) | 0.59 | (0.21-1.65) |  |  |  |  |  |  |  |
| **Sexual preference^2^** |  |  |  |  |  |  |  |  |  |  |  |
| Exclusively homosexual | **REF** |  | **REF** |  | **0.0451** |  |  |  |  |  |  |
| Not exclusively homosexual | **0.35** | **(0.15-0.80)** | **0.54** | **(0.21-1.34)** |  |  |  |  |  |  |  |
| **Post-exposure prophylaxis used (6M)^3^** |  |  |  |  | 0.304 |  |  |  |  |  |  |
| No | REF |  | REF |  |  |  |  |  |  |  |  |
| Yes | 1.40 | (0.51-3.87) | 0.53 | (0.14-2.11) |  |  |  |  |  |  |  |
| **Sexually transmitted infection (6M)^3,4^** |  |  |  |  |  |  |  |  |  |  |  |
| No | REF |  | REF |  | 0.472 |  |  |  |  |  |  |
| Yes | 1.52 | (0.69-3.38) | 1.51 | (0.71-3.18) |  |  |  |  |  |  |  |
| **Alcohol use disorder identification test (AUDIT)^2,5^** |  |  |  |  |  |  |  |  |  |  |  |
| Score <8 | REF |  | REF |  | 0.861 |  |  |  |  |  |  |
| Score ≥8 | 1.24 | (0.54-2.83) | 1.18 | (0.54-2.59) |  |  |  |  |  |  |  |
| **Drug use disorder identification test (DUDIT)^2,5^** |  |  |  |  |  |  |  |  |  |  |  |
| Score <8 | REF |  | REF |  | 0.462 |  |  |  |  |  |  |
| Score ≥8 | 0.71 | (0.34-1.45) | 1.08 | (0.56-2.08) |  |  |  |  |  |  |  |

**Abbreviations:** AMPrEP, Amsterdam PrEP demonstration project; aOR, adjusted odds ratio; CAS, condomless anal sex; CI, confidence interval; PrEP, pre-exposure prophylaxis; OR, odds ratio

Note 1: The OR should be interpreted as the odds ratio for the 1 unit increase of the determinant or the switching from the reference category to another category for the outcome (1-26 days or ≤27 days) versus the reference outcome (0 days app use). For example, the OR per 1 month increase since PrEP initiation is 0.97 for using the app 1-26 days vs using the app 0 days. In other words, the expected odds of using the app 1-26 days is lower with longer time since PrEP initiation.

Note 2: gender was not taken into account as none of the transgender women using event-driven PrEP used the app during the study period.

1. Time-updated
2. As reported at baseline
3. In the 6 months before baseline
4. At least one bacterial sexually transmitted infection (i.e. syphilis, or urethral or rectal chlamydia or gonorrhoea)
5. A score of eight or higher indicates possible presence of alcohol- or drug-use disorder**Supplementary Table 2: Comparison of the numbers of PrEP pills taken and unknown casual partners reported in the AMPrEP mobile diary app and the 3-monthly questionnaires, AMPrEP observational cohort study, September 2015 to October 2019, Amsterdam, the Netherlands.**

|  |  | **App data** | | **Questionnaire data** | |  |
| --- | --- | --- | --- | --- | --- | --- |
|  | *N* | *Median* | *IQR* | *Median* | *IQR* | *p-value* |
| **Number of pills^1^** |  |  |  |  |  |  |
| 3 months | 54 | 12 | [8-19] | 13 | [9-20] | 0.598 |
| 6 months | 41 | 14 | [8-21] | 14 | [9-20] | 0.590 |
| 9 months | 44 | 13 | [5-20] | 16 | [7-24] | 0.0275 |
| 12 months | 35 | 9 | [2-17] | 11 | [2-17] | 0.129 |
| 15 months | 38 | 13 | [6-21] | 14 | [7-22] | 0.952 |
| 18 months | 40 | 12 | [4-19] | 20 | [9-22] | 0.0020 |
| 21 months | 38 | 11 | [6-19] | 13 | [8-19] | 0.327 |
| 24 months | 34 | 7 | [0-15] | 10 | [4-14] | 0.0414 |
| 27 months | 31 | 9 | [2-19] | 11 | [4-20] | 0.474 |
| 30 months | 30 | 9 | [2-18] | 14 | [6-19] | 0.0015 |
| 33 months | 28 | 9 | [2-14] | 10 | [3-17] | 0.361 |
| 36 months | 36 | 11 | [4-15] | 13 | [5-18] | 0.0224 |
| 39 months | 32 | 9 | [4-17] | 13 | [8-18] | 0.0159 |
| 42 months | 13 | 12 | [5-15] | 12 | [6-17] | 0.775 |
| **Number of unknown casual partners^2^** |  |  |  |  |  |  |
| 3 months | 48 | 3 | [1-6] | 3 | [1-9] | 0.0118 |
| 6 months | 45 | 2 | [0-4] | 3 | [1-8] | 0.0017 |
| 9 months | 40 | 2 | [0-7] | 3 | [0-9] | 0.0099 |
| 12 months | 36 | 2 | [1-5] | 2 | [0-8] | 0.255 |
| 15 months | 35 | 2 | [0-6] | 3 | [0-9] | 0.0032 |
| 18 months | 38 | 1 | [0-4] | 1 | [0-6] | 0.0015 |
| 21 months | 39 | 1 | [0-4] | 1 | [0-5] | 0.306 |
| 24 months | 36 | 1 | [0-3] | 2 | [0-3] | 0.0160 |
| 27 months | 36 | 1 | [0-4] | 1 | [0-5] | 0.0137 |
| 30 months | 30 | 1 | [0-3] | 1 | [0-5] | 0.0542 |
| 33 months | 27 | 1 | [0-5] | 2 | [0-15] | 0.0007 |
| 36 months | 36 | 1 | [0-3] | 2 | [0-5] | 0.0277 |
| 39 months | 32 | 1 | [0-3] | 1 | [0-6] | 0.062 |
| 42 months | 13 | 0 | [0-2] | 0 | [0-3] | 0.0465 |

**Abbreviations:** AMPrEP, Amsterdam PrEP demonstration project; IQR, interquartile range; UCP, unknown casual partner

1. In the 30 days before the questionnaire

2. In the 90 days before the questionnaire

NB: In the app participants could only indicate whether they had taken pills (yes/no), but not the number of pills. We assumed that on the first day of a PrEP course participants took two pills and all subsequent days of that course one pill. In the table this derived number of pills taken based on app data is compared to the reported number of pills taken according to the questionnaire.

**Supplementary** **Table 3: Socio-demographic and sexual behaviour characteristics at baseline of event-driven PrEP users who ever used the app (n=141) and never used the app (n=41), AMPrEP observational cohort study, August 2015 to June 2016, Amsterdam, the Netherlands.**

|  | **Included in the analysis** | | **Excluded from the analysis** | |  |
| --- | --- | --- | --- | --- | --- |
|  | **(n=141)** | | **(n=41)** | | *p-value* |
|  | *n^1^* | *%^1^* | *n^1^* | *%^1^* |  |
| **Age (years)** |  |  |  |  |  |
| Median (IQR) | 42 | [33-50] | 38 | [33-51] | *0*.*780* |
| <35 year | 46 | 33% | 14 | 34% | *0*.*961* |
| 35 - 44 year | 40 | 28% | 12 | 29% |  |
| ≥45 year | 55 | 39% | 15 | 37% |  |
| **Gender identity** |  |  |  |  |  |
| Male | 141 | 100% | 40 | 99% | *0*.*225* |
| Transgender woman | 0 | 0% | 1 | 1% |  |
| **Self-declared ethnicity** |  |  |  |  |  |
| White | 123 | 87% | 33 | 80% | *0*.*277* |
| Non-white | 18 | 13% | 8 | 20% |  |
| **Residence** |  |  |  |  |  |
| Amsterdam | 94 | 67% | 30 | 73% | *0*.*431* |
| Other | 47 | 33% | 11 | 27% |  |
| **Highest education level** |  |  |  |  |  |
| No college/university | 22 | 16% | 11 | 27% | *0*.*101* |
| College/university | 119 | 84% | 30 | 73% |  |
| **Employment** |  |  |  |  |  |
| Employed | 113 | 80% | 30 | 73% | *0*.*338* |
| Unemployed | 28 | 20% | 11 | 27% |  |
| **Monthly net income** |  |  |  |  |  |
| Low (≤€1700) | 35 | 26% | 15 | 41% | *0.0114* |
| Middle (€1701 to €2950) | 53 | 39% | 18 | 49% |  |
| High (>€2950) | 49 | 36% | 4 | 11% |  |
| **Steady relationship** |  |  |  |  |  |
| No | 72 | 52% | 30 | 73% | *0*.*0152* |
| Yes | 67 | 48% | 11 | 27% |  |
| **Living situation** |  |  |  |  |  |
| Alone | 78 | 55% | 26 | 63% | *0*.*371* |
| With partner | 47 | 33% | 9 | 22% |  |
| With others | 16 | 11% | 6 | 15% |  |
| **Sexual preference** |  |  |  |  |  |
| Exclusively homosexual | 114 | 81% | 26 | 65% | *0*.*0345* |
| Not exclusively homosexual | 27 | 19% | 14 | 35% |  |
| **Number of anal sex partners (3M)^2^** | |  |  |  |  |
| Median (IQR) | 9 | [4-18] | 6 | [3-20] | *0*.*463* |
| **CAS with casual partner (6M)^3^** |  |  |  |  |  |
| No | 5 | 4% | 2 | 5% | *0*.*656* |
| Yes | 136 | 96% | 39 | 95% |  |
| **Post-exposure prophylaxis used (6M)^3^** | |  |  |  |  |
| No | 129 | 91% | 41 | 100% | *0*.*071* |
| Yes | 12 | 9% | 0 | 0 |  |
| **HIV-positive partner with a detectable viral load (6M)^3^** | |  |  |  |  |
| No | 141 | 100% | 39 | 95% | *0*.*0498* |
| Yes | 0 | 0% | 2 | 5% |  |
| **Sexually transmitted infection (6M)^3,4^** | |  |  |  |  |
| No | 89 | 63% | 30 | 73% | *0*.*234* |
| Yes | 52 | 37% | 11 | 27% |  |
| **Indication of hazardous alcohol-use (AUDIT)^5^** |  |  |  |  |  |
| Score <8 | 98 | 71% | 32 | 78% | *0*.*375* |
| Score ≥8 | 40 | 29% | 9 | 22% |  |
| **Indication of hazardous drug-use (DUDIT)^5^** |  |  |  |  |  |
| Score <8 | 90 | 65% | 26 | 63% | *0*·*832* |
| Score ≥8 | 48 | 35% | 15 | 37% |  |

**Abbreviations:** AMPrEP, Amsterdam PrEP demonstration project; CAS, condomless anal sex act; HIV, human immunodeficiency virus; IQR, interquartile range; PrEP, pre-exposure prophylaxis

Data were missing for income (n=8), steady relationship at baseline (n=2), sexual preference (n=1), AUDIT (n=3), DUDIT (n=3).

1. Unless stated otherwise
2. In the 3 months before baseline
3. In the 6 months before baseline
4. At least one bacterial sexually transmitted infection (i.e. syphilis, urethral or rectal chlamydia or gonorrhoea)
5. A score of eight or higher indicates possible presence of alcohol- or drug-use disorder

**Supplementary Table 4: PrEP protection of condomless anal sex acts by edPrEP users per partner type as reported in the daily app by participants who reported data in the app at least 27 days within a month (n=108), AMPrEP observational cohort study, September 2015 to October 2019, Amsterdam, the Netherlands.**

|  | **Steady partner**  **(n=1,913)** | | **Known casual partner**  **(n=2,758)** | | **Unknown casual partner**  **(n=1,805)** | |
| --- | --- | --- | --- | --- | --- | --- |
|  | n | % | n | % | n | % |
| **PrEP protection^1^** |  |  |  |  |  |  |
| None | 545 | 28.5% | 108 | 3.9% | 73 | 4.0% |
| Poor | 162 | 8.5% | 71 | 2.6% | 79 | 4.4% |
| Good | 182 | 9.5% | 190 | 6.9% | 135 | 7.5% |
| Excellent | 1,024 | 53.5% | 2,389 | 86.6% | 1,518 | 84.1% |

**Abbreviations:** PrEP, pre-exposure prophylaxis

1. Definitions of PrEP coverage in Box 1.

**Supplementary Table 5: Determinants of poor/no adherence to PrEP per sex partner type among event-driven PrEP users (n=141); results of univariable multi-level logistic regression, AMPrEP observational cohort study, Septebmber 2015 to October 2019, Amsterdam, the Netherlands.**

|  | **CAS days with steady partners**  **(n=2,455)** | | | **CAS days with known casual partners**  **(n=3,472)** | | | **CAS days with unknown casual partners**  **(n=2,297)** | | |
| --- | --- | --- | --- | --- | --- | --- | --- | --- | --- |
|  | *OR* | *95% CI* | *p-value* | *OR* | *95% CI* | *p-value* | *OR* | *95% CI* | *p-value* |
| **Months since PrEP initiation** | 1.02 | (1.00-1.03) | 0.0093 | **1.02** | **(1.01-1.04)** | **0.0016** | 1.01 | (0.99-1.03) | 0.344 |
| **Age, per 10 year increase^1^** | 0.92 | (0.53-1.59) | 0.753 | **0.73** | **(0.54-0.98)** | **0.0392** | 0.74 | (0.53-1.04) | 0.085 |
| **Age^1^** |  |  |  |  |  |  |  |  |  |
| <35 year | REF |  | 0.471 | **REF** |  | **0.0033** | REF |  | 0.086 |
| 35 - 44 year | 0.42 | (0.11-1.68) |  | **0.25** | **(0.11-0.58)** |  | 0.43 | (0.20-0.95) |  |
| ≥45 year | 0.44 | (0.11-1.73) |  | **0.35** | **(0.16-0.76)** |  | 0.48 | (0.20-1.14) |  |
| **Self-declared ethnicity^2^** |  |  |  |  |  |  |  |  |  |
| White | REF |  | 0.954 | REF |  | 0.525 | REF |  | 0.730 |
| Non-white | 1.06 | (0.15-7.45) |  | 1.42 | (0.48-4.25) |  | 1.25 | (0.35-4.47) |  |
| **Residence^2^** |  |  |  |  |  |  |  |  |  |
| Amsterdam | REF |  | 0.328 | REF |  | 0.686 | REF |  | 0.737 |
| Other | 1.90 | (0.52-6.89) |  | 0.85 | (0.39-1.85) |  | 0.86 | (0.34-2.13) |  |
| **Highest education level^2^** |  |  |  |  |  |  |  |  |  |
| No college/university | REF |  | 0.541 | REF |  | 0.127 | REF |  | 0.109 |
| College/university | 0.55 | (0.08-3.80) |  | 0.47 | (0.17-1.24) |  | 0.43 | (0.15-1.21) |  |
| **Employment^2^** |  |  |  |  |  |  |  |  |  |
| Employed | REF |  | 0.261 | REF |  | 0.727 | REF |  | 0.215 |
| Unemployed | 2.83 | (0.46-17.30) |  | 1.19 | (0.44-3.20) |  | 0.49 | (0.16-1.51) |  |
| **CAS with a steady partner^1,3^** |  |  |  |  |  |  |  |  |  |
| No | NA |  |  | REF |  | 0.132 | REF |  | 0.940 |
| Yes | NA |  |  | 1.39 | (0.91-2.12) |  | 0.98 | (0.59-1.63) |  |
| **Living situation^2^** |  |  |  |  |  |  |  |  |  |
| Alone | REF |  | 0.073 | **REF** |  | **0.0130** | REF |  | 0.152 |
| With partner | 0.71 | (0.20-2.52) |  | **0.39** | **(0.17-0.87)** |  | 0.43 | (0.17-1.07) |  |
| With others | 6.60 | (0.96-45.44) |  | **1.93** | **(0.63-6.02)** |  | 1.17 | (0.29-4.65) |  |
| **Sexual preference^2^** |  |  |  |  |  |  |  |  |  |
| Exclusively homosexual | REF |  | 0.525 | REF |  | 0.148 | REF |  | 0.339 |
| Not exclusively homosexual | 1.64 | (0.36-7.44) |  | 2.07 | (0.77-5.55) |  | 1.76 | (0.55-5.59) |  |
| **Post-exposure prophylaxis used (6M)^4^** | |  |  |  |  |  |  |  |  |
| No | REF |  | 0.168 | REF |  | 0.706 | REF |  | 0.971 |
| Yes | 0.21 | (0.02-1.92) |  | 1.36 | (0.28-6.65) |  | 1.03 | (0.20-5.29) |  |
| **Sexually transmitted infection (6M)^4,5^** | |  |  |  |  |  |  |  |  |
| No | REF |  | 0.540 | REF |  | 0.788 | REF |  | 0.188 |
| Yes | 0.68 | (0.19-2.36) |  | 1.11 | (0.52-2.39) |  | 1.79 | (0.75-4.28) |  |
| **Alcohol use disorder identification test (AUDIT)^1,6^** | | |  |  |  |  |  |  |  |
| Score <8 | REF |  | 0.286 | **REF** |  | **0.0346** | REF |  | 0.328 |
| Score ≥8 | 0.60 | (0.24-1.53) |  | **1.98** | **(1.05-3.74)** |  | 1.40 | (0.72-2.72) |  |
| **Drug use disorder identification test (DUDIT)^1,6^** | | |  |  |  |  |  |  |  |
| Score <8 | **REF** |  | **0.0037** | REF |  | 0.327 | REF |  | 0.534 |
| Score ≥8 | **0.42** | **(0.17-0.76)** |  | 0.77 | (0.46-1.30) |  | 0.85 | (0.50-1.44) |  |

**Abbreviations:** AMPrEP, Amsterdam PrEP demonstration project; CAS, condomless anal sex; CI, confidence interval; NA, not applicable; OR, odds ratio; PrEP, pre-exposure prophylaxis

1. Time-updated
2. As reported at baseline
3. In the 30 days before CAS with a known or unknown casual partner, respectively
4. In the 6 months before baseline
5. At least one bacterial sexually transmitted infection (i.e. syphilis, or urethral or rectal chlamydia or gonorrhoea)
6. A score of eight or higher indicates possible presence of alcohol- or drug-use disorder
7. Univariable analysis not performed due to 0 observations in one of the cells

**Supplementary** **Figure 1.** **App use after PrEP initiation among event-driven PrEP users (n=182), AMPrEP observational cohort study, September 2015 to October 2019, Amsterdam, the Netherlands.**

**
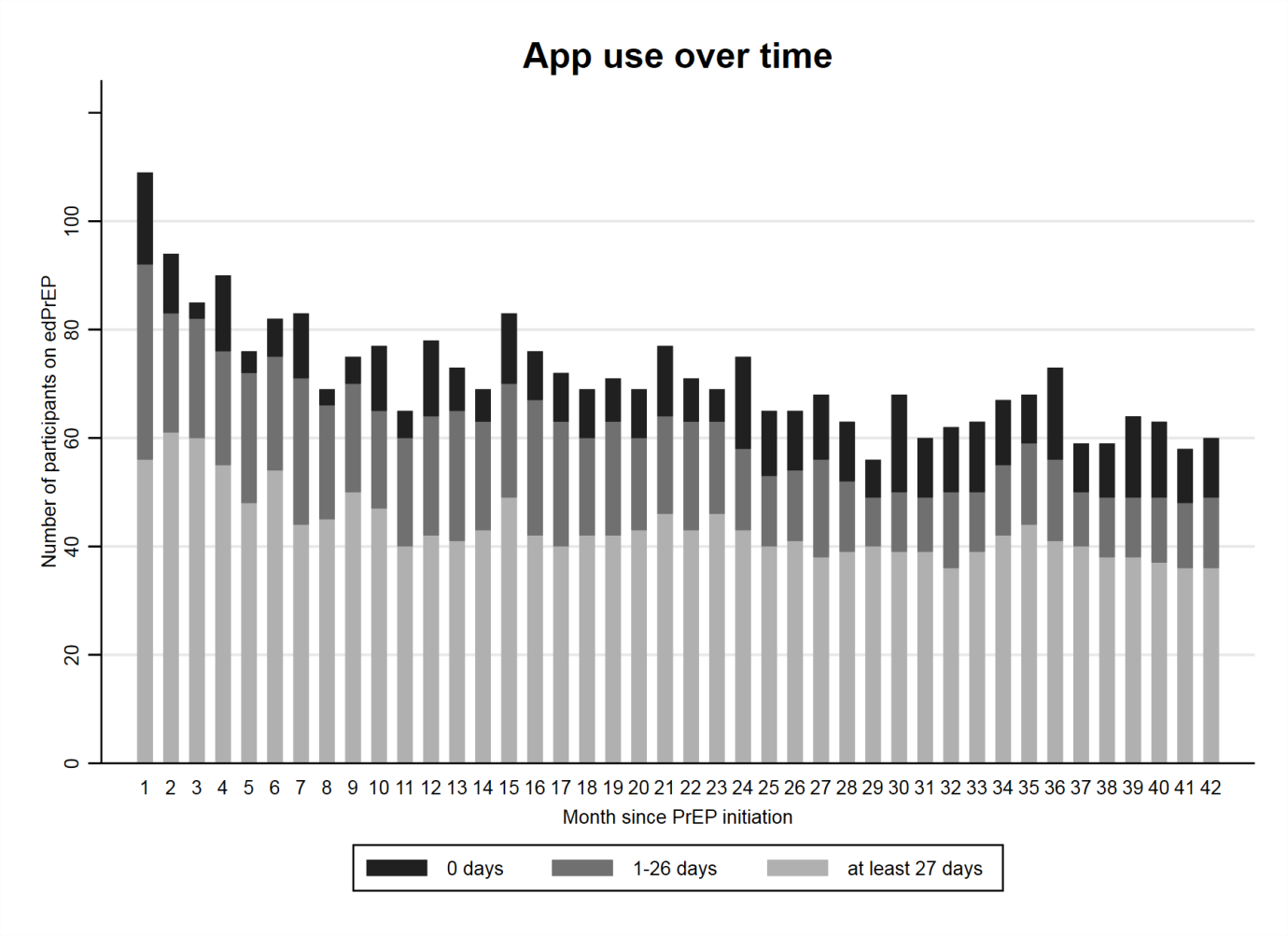
Supplementary Figure 2: Association between log transformed TFV-DP concentrations from dried blood spots (DBS) and log transformed number of pills taken as reported in the 3-monthly questionnaires, AMPrEP observational cohort study, September 2015 to October 2019, Amsterdam, the Netherlands. Number of pills was transformed as follows: ln(1+number of pills taken)**

*
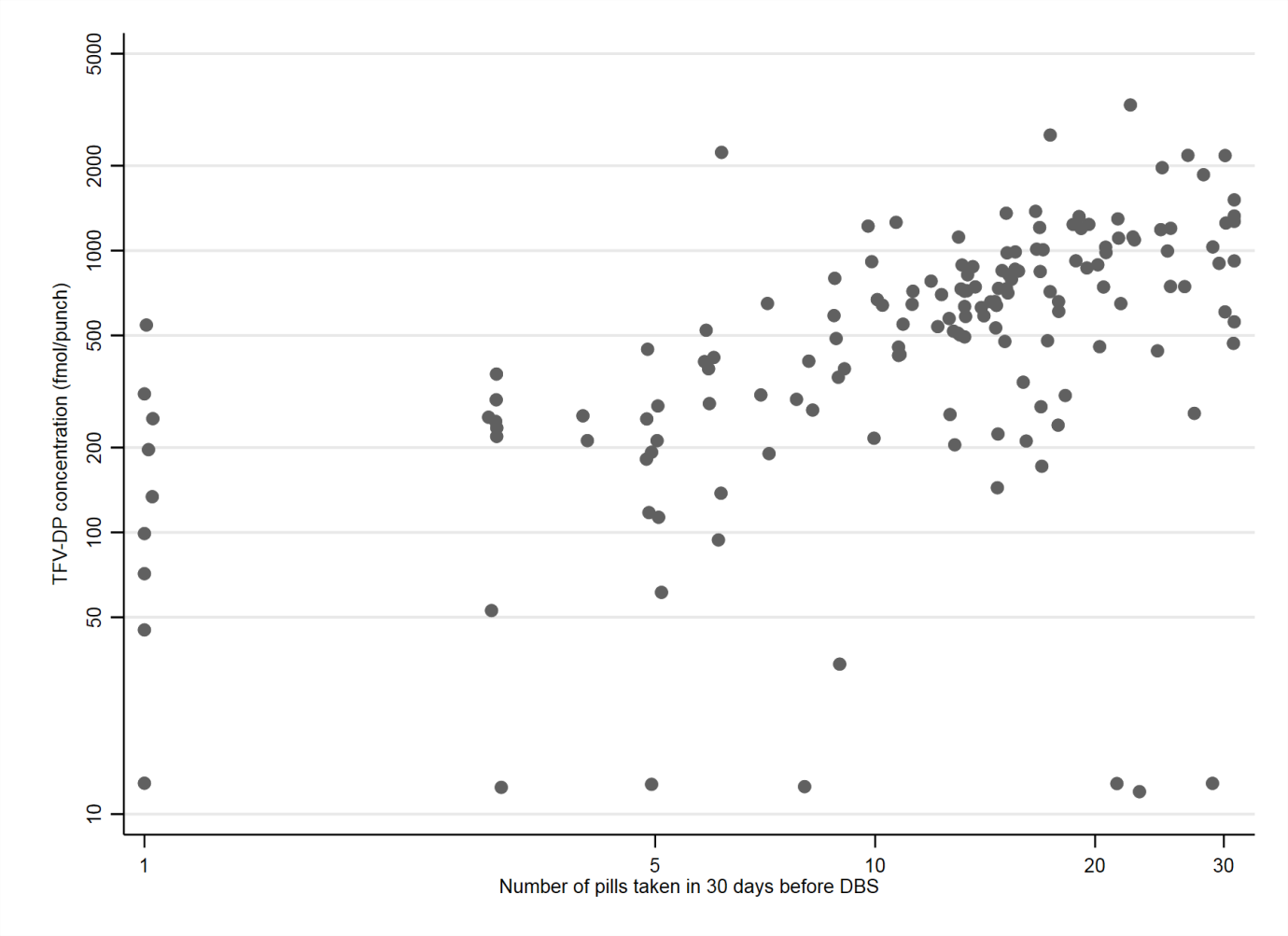
*

# Annex S1: Acknowledgements of the H-TEAM consortium

**H-TEAM Steering Committee:**

J.E.A.M. van Bergen^6;4;5^, G.J. de Bree^1;2^, P. Brokx^8^, F. Deug^6^, M. Heidenrijk^1^, M. Prins^3;2^, P. Reiss^1;7^ (chair), M. van der Valk^2^

**H-TEAM Core Project Group:**

J.E.A.M. van Bergen^6;4;5^, G.J. de Bree^1;2^ (chair), P. Brokx^8^, U. Davidovich^3^, S.E. Geerlings^2^, E. Hoornenborg^3^, A. Oomen^6^, A. van Sighem^7^, W. Zuilhof^6^ H-TEAM Project Management: M.L. Groot Bruinderink^1^

**H-TEAM additional collaborators:**

R.C.A. Achterbergh^3^, M. van Agtmael^24^, J. Ananworanich^22^, D. Van de Beek^17^, G.E.L. van den Berk^11^, D. Bezemer^7^, A. van Bijnen^6^, W.L. Blok^11^, S. Bogers^2^, M. Bomers^24^, C.A.B. Boucher^13^, W. Brokking^26^, D. Burger^20^, K. Brinkman^11^, N. Brinkman^32^, M. de Bruin^12^, S. Bruisten^3^, L. Coyer^3^, R. van Crevel^29^, C.G. Daans^3;34^, L. Dellemann^6^, M. Dijkstra^3^, Y.T. van Duijnhoven^3^, A. van Eeden^26^, L. Elsenburg^26^, M.A.M. van den Elshout^3^, C. Ester^7^, E. Ersan^3^, P. E.V. Felipa^3^, P.H.J. Frissen^11^, T.B.H. Geijtenbeek^18^, M.H. Godfried^2^, J. van Gool^3^, A. Goorhuis^2^, M. Groot^26^, C.A. Hankins^1^, A. Heijnen^30;31^, M.M.J Hillebregt^7^, A. Hogewoning^3^, M. Hommenga^3^, J.W. Hovius^2^, Y. Janssen^32^, K. de Jong^3^, V. Jongen^3^, N.A. Kootstra^19^, R.A. Koup^21^, F.P.  Kroon^16^, T.J.W. van de Laar^35;36^, F. Lauw^37^, M. M. van Leeuwen^3^, K. Lettinga^27^, I. Linde^3^, D.S.E. Loomans^3^, J.T. van der Meer^2^, T. Mouhebati^6^, B.J. Mulder^3^, J. Mulder^25^, F.J. Nellen^2^, A. Nijsters^6^, H. Nobel^2^, P. Oostvogel^3^, E.L.M. Op de Coul^5^, E. Peters^24^, I.S. Peters^3^, T. van der Poll^2^, O. Ratmann^28^, C. Rokx^14^, M.S. van Rooijen^3^, M.F. Schim van der Loeff^3;10^, W.E.M. Schoute^11^, G.J. Sonder^3^, J. Veenstra^27^, A. Verbon^14^, F. Verdult^8^, J. de Vocht^24^, H.J. de Vries^3;9;10^, S. Vrouenraets^27^, M. van Vugt^2^, W.J. Wiersinga^2^, F.W. Wit^2;7^, L.R. Woittiez^2^, S. Zaheri^7^, P. Zantkuijl^6^, M.C. van Zelm^23^, A. Żakowicz^33^, H.M.L. Zimmermann^3^, K. Yap^3^.

**Affiliations:**

1 Department of Global Health, Amsterdam UMC – location AMC, and Amsterdam Institute for Global Health and Development, Amsterdam, the Netherlands

2 Department of Internal Medicine, Division of Infectious Diseases, Amsterdam UMC – location AMC, Amsterdam, the Netherlands

3 Department of Infectious Diseases, Public Health Service of Amsterdam, Amsterdam, the Netherlands

4 Department of General Practice, Amsterdam UMC – location AMC, University of Amsterdam, Amsterdam, the Netherlands

5 Epidemiology and Surveillance Unit, Center for Infectious Disease Control, National Institute of Public Health and the Environment, the Netherlands

6 STI AIDS Netherlands, Amsterdam, the Netherlands

7 Stichting HIV Monitoring, Amsterdam, the Netherlands

8 Dutch Association of PLHIV, Amsterdam, the Netherlands

9 Department of Dermatology, Amsterdam UMC – location AMC, University of Amsterdam, Amsterdam, the Netherlands

10 Amsterdam institute for Infection & Immunity (AII), Amsterdam UMC – location AMC, University of Amsterdam, Amsterdam, the Netherlands

11 Department of internal medicine, OLVG – location East, Amsterdam, the Netherlands

12 Aberdeen Health Psychology Group, Institute of Applied Health Sciences, University of Aberdeen, Aberdeen, United Kingdom

13 Department of viro-science, Erasmus Medical Center Rotterdam, Rotterdam, the Netherlands

14 Department of Internal Medicine and Infectious Diseases, Erasmus Medical Center, Rotterdam, the Netherlands

16 Department of Infectious Diseases, Leiden University Medical Center, Leiden, the Netherlands

17 Amsterdam institute for Infection & Immunity (AII), Department of Neurology, Amsterdam UMC – location AMC, Amsterdam, the Netherlands

18 Laboratory of Experimental Immunology, Amsterdam UMC – location AMC Amsterdam, the Netherlands

19 Laboratory for Viral Immune Pathogenesis, Amsterdam UMC – location AMC Amsterdam, the Netherlands

20 Department of Pharmacy, Radboud University Nijmegen Medical Center, Nijmegen, the Netherlands

21 Immunology Laboratory, Vaccine Research Center, NIAID, National Institutes of Health

22 US Military HIV Research Program and the Henry M. Jackson Foundation for the Advancement of Military Medicine, Bethesda, United States

23 Department of Virology, Erasmus Medical Center, Rotterdam, the Netherlands

24 Department of Internal Medicine, Amsterdam UMC – location VUMC, Amsterdam, the Netherlands

25 Department of Internal Medicine, Slotervaart Hospital, Amsterdam, the Netherlands

26 DC Clinics, Amsterdam, the Netherlands

27 Department of Internal Medicine, OLVG – location West , Amsterdam, the Netherlands

28 School of Public Health, Faculty of Medicine, Imperial College London, London, United Kingdom

29 Department of Internal Medicine, Radboud University Nijmegen Medical Center, Nijmegen, the Netherlands

30 Sexology Center Amsterdam, Amsterdam, the Netherlands

31 GP practice Heijnen & de Meij, Amsterdam, the Netherlands

32 Elaa – First line Amsterdam Almere, Amsterdam, the Netherlands

33 AIDS Healthcare Foundation, Amsterdam, the Netherlands

34 Center of Expertise on Gender Dysphoria, Amsterdam UMC – location VUMC, Amsterdam, the Netherlands

35 Department of Medical Microbiology, OLVG, Amsterdam, the Netherlands

36 Department of Donor Medicine Research, Laboratory of Blood-borne Infections, Sanquin Research, Amsterdam, the Netherlands

37 Department of Internal Medicine, Medical Center Jan van Goyen, Amsterdam, the Netherlands
